# Supplementary material for: In situ observation of coalescence of nuclei in colloidal crystal-crystal transitions
Source: Nat Commun. 2023 Aug 15;14:4905. doi: 10.1038/s41467-023-40627-w (PMC10427646; doi:10.1038/s41467-023-40627-w)
Supplement: Supplementary file 1 — Supplementary Information [file 41467_2023_40627_MOESM1_ESM.pdf]

Supplementary Information for

# In situ observation of coalescence of nuclei in colloidal crystal-crystal transitions

Yi Peng[1,2,3*], Wei Li[2], Tim Still[4], Arjun G. Yodh[4] and Yilong Han[2*]

[1*]Beijing National Laboratory for Condensed Matter Physics, Institute of Physics, Chinese Academy of Sciences, Beijing 100190, China.

[2*]Department of Physics, Hong Kong University of Science and Technology, Hong Kong, China.

[3]School of Physical Sciences, University of Chinese Academy of Sciences, Beijing 100049, China.

[4]Department of Physics and Astronomy, University of Pennsylvania, Philadelphia, Pennsylvania 19104, USA.

*Corresponding author(s). E-mail(s): pengy@iphy.ac.cn; yilong@ust.hk;

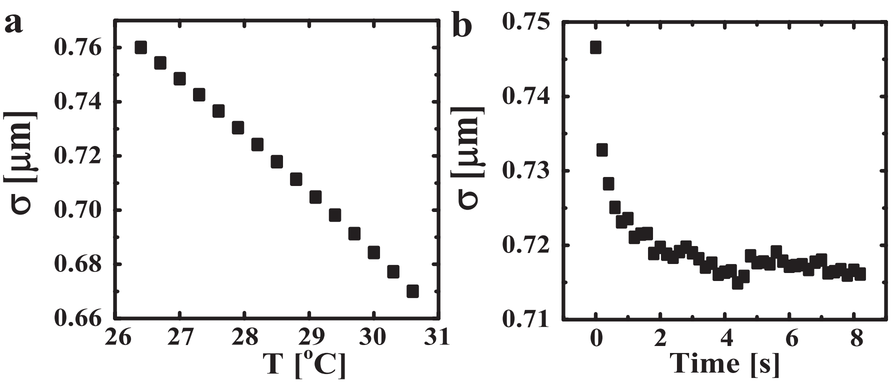


**Supplementary Fig. 1 Characterization of colloidal particles. a**, Mean effective diameter of isolated NIPA microgel spheres stuck onto a glass slide decreases with increased temperature. **b**, Mean diameter of particles stuck onto a glass slide decreases in the first 3 s after switching on the heating light at $t = 0$ s. The figures are reproduced from ref. [1]. Reprinted with permission from AAAS.

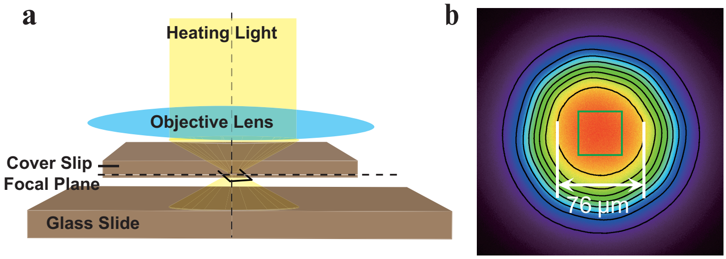


**Supplementary Fig. 2 Experimental setup for local heating. a**, Schematic of local optical heating. **b**, Measured temperature profile in the $xy$ plane. The contour spacing is 0.2°C. The temperature difference in the $\pi(38\mu\text{m})^2$ area of the central circle is less than 0.2°C, which is larger than the field of view (green box).

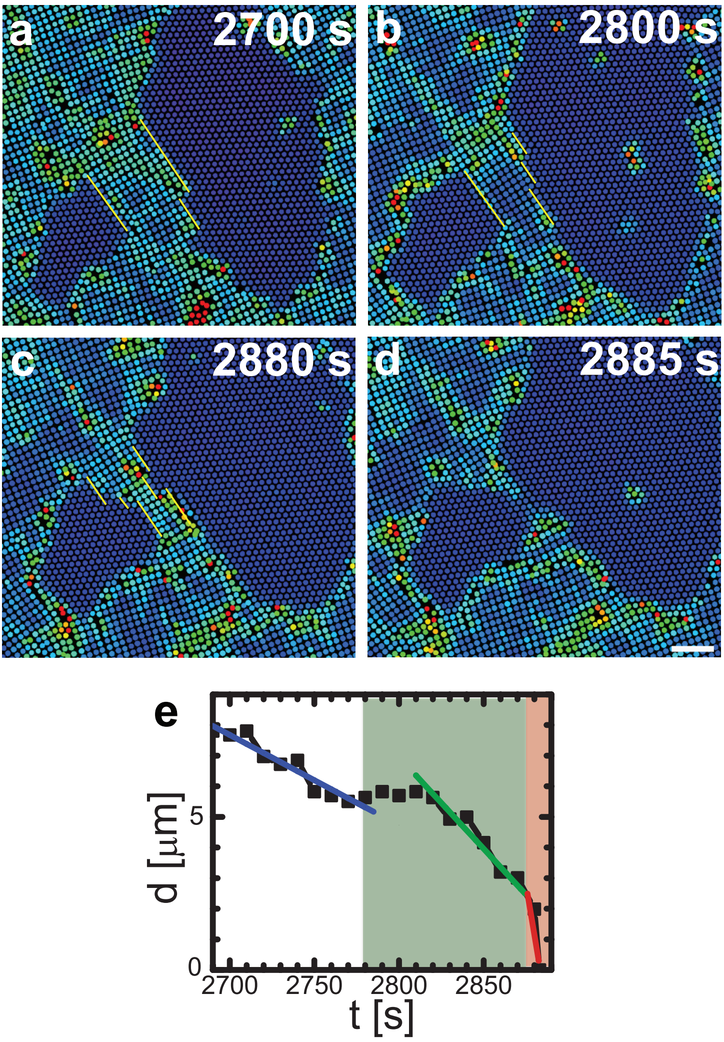


**Supplementary Fig. 3 Typical coalescence of two large △-lattice nuclei with coherent □-△ interfaces.** Particles are coloured by their Lindemann parameters, as shown in Fig. 1. **a**, At $t = 2700$ s, two nuclei whose closest surfaces (yellow lines) and their [01] lattice directions are all parallel to each other. These surfaces are coherent interfaces. **b**, The coherent interfaces approach to each other by ledge mechanisms, compressing □ lattice between them when the separation is around the critical distance $\sim 6$ particles. The Lindemann parameters of the □ lattice decrease, as shown by the blue particles between yellow line segments, and the approaching speed reduces to zero within 2770−2810 s. **c**, After a temporary stop, new ledges and kinks develop on the coherent interfaces, which rotates the □ lattice and reduces the coherency. The dropped Lindemann parameters increase, and the nuclei grow faster than before. **d**, The expansion of two nuclei distort the □ lattice to △ lattice, fusing the two nuclei together at $t = 2885$ s. **e**, The separation between two nuclei decreases with time. The green and red regions represent the stages that nuclei grow faster and merge together, respectively. Scale bar: 5 $\mu$m.

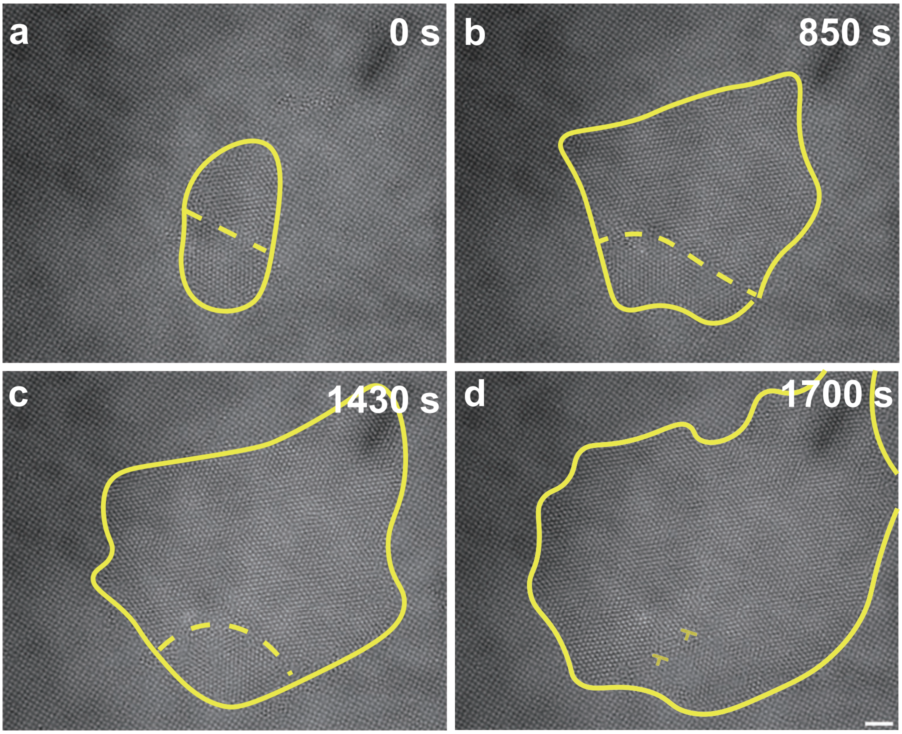


**Supplementary Fig. 4 The migration of a GB after two nuclei coalesced.** **a**, Two nuclei coalesce and form a grain boundary between them. Solid curves indicate the interface between two phases, and dashed curves the GBs. **b**, The upper grain of product phase grow faster and the lower one slower. **c**, The GB between two grains move the the smaller grain. **d**, The smaller nucleus rotates, the GB disappears and two dislocations ($\perp$) are left. Scale bar: 5 $\mu$m.

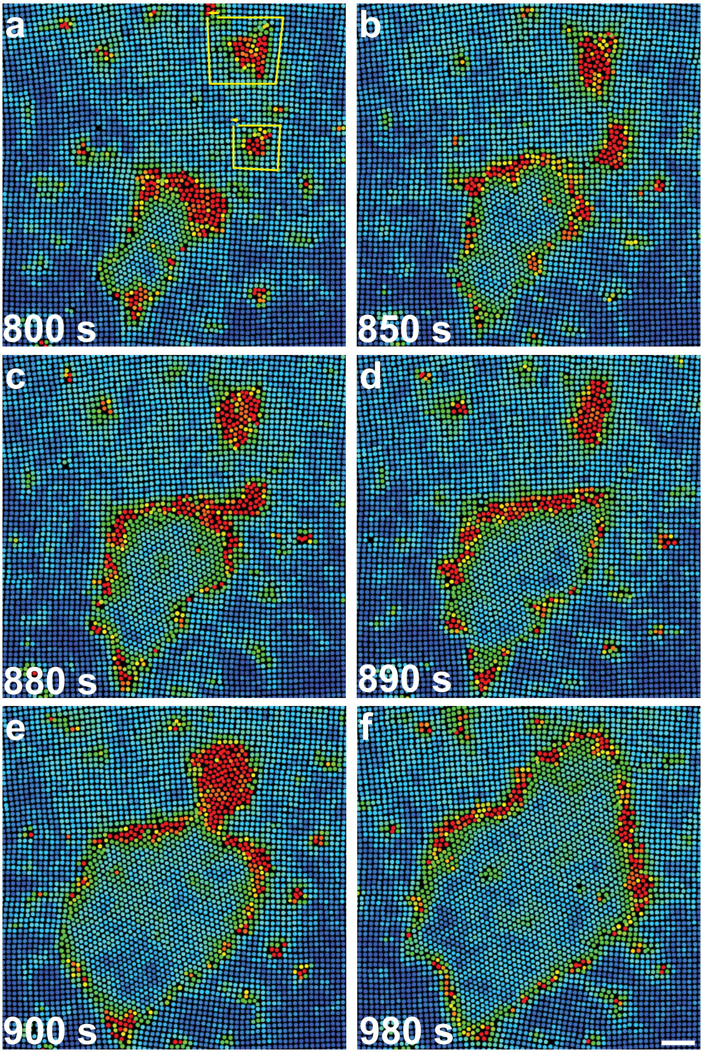


**Supplementary Fig. 5 Two small liquid nuclei absorbed by a large △-lattice nucleus.** Particles are coloured by their Lindemann parameter shown in Fig. 1. **a**, Two metastable liquid nuclei form at the dislocations, signified by open Burgers circuits (yellow loops) and Burgers vectors (yellow arrows). **b**, They are elongated when the growth of the large △-lattice nucleus changes the nearby strain field. **c**, The first liquid nucleus is absorbed into the △-lattice nucleus and (**d**) recrystallises, and then (**e**) the second liquid nucleus is absorbed and (**f**) recrystallises. Scale bar: 5 $\mu$m.

## References

[1] Wang, Z., Wang, F., Peng, Y., Zheng, Z. & Han, Y. Imaging the homogeneous nucleation during the melting of superheated colloidal crystals. *Science* **338**, 87–90 (2012).
