# Supplementary material for: In situ observation of coalescence of nuclei in colloidal crystal-crystal transitions
Source: Nat Commun. 2023 Aug 15;14:4905. doi: 10.1038/s41467-023-40627-w (PMC10427646; doi:10.1038/s41467-023-40627-w)
Supplement: Supplementary file 3 — Description of Additional Supplementary Files [file 41467_2023_40627_MOESM3_ESM.pdf]

Description of Additional Supplementary Files

Supplementary Movie 1: Coalescence between two triangle-lattice nuclei inside a crystalline domain shown in Figure 1. The left part is the raw video, and the right part is the same video but with the colour-coded Lindemann parameters. Colour bar is the same as that in Fig. 1. Scale bar: 5 μm. 25X real time.

Supplementary Movie 2: Coalescence between a large △-lattice nucleus and a small one inside a crystalline domain shown in Figure 3. The small one liquefies when it is closed to the large one. The left part is the raw video, and the right part is the same video but with the colour-coded Lindemann parameters. Colour bar is the same as that in Fig. 1. Scale bar: 5 μm. 10X real time.

Supplementary Movie 3: Coalescence between two △-lattice nuclei on a low-angle grain boundary shown in Figure 4. The left part is the raw video, and the right part is the same video but with the colour-coded Lindemann parameters. Colour bar is the same as that in Fig. 1. Scale bar: 5 μm. 25 X real time.

Supplementary Movie 4: Coalescence between two triangle-lattice nuclei on a high-angle grain boundary shown in Figure 5. The left part is the raw video, and the right part is the same video but with the colour-coded Lindemann parameters. Colour bar is the same as that in Fig. 1. Scale bar: 5 μm. 25 X real time.
